# Current Evidence on the Protective Effects of Recombinant Human Erythropoietin and Its Molecular Variants against Pathological Hallmarks of Alzheimer’s Disease

**DOI:** 10.3390/ph13120424

**Published:** 2020-11-26

**Authors:** José J. Jarero-Basulto, Martha C. Rivera-Cervantes, Deisy Gasca-Martínez, Francisco García-Sierra, Yadira Gasca-Martínez, Carlos Beas-Zárate

**Affiliations:** 1 Cellular Neurobiology Laboratory, Cell and Molecular Biology Department, CUCBA, University of Guadalajara, Zapopan 45220, Mexico; 2 Behavioral Analysis Unit, Neurobiology Institute, Campus UNAM-Juriquilla, Querétaro 76230, Mexico; gasca@inb.unam.mx; 3 Department of Cell Biology, Center of Research and Advanced Studies of the National Polytechnic Institute (CINVESTAV), Ciudad de Mexico 07360, Mexico; fgarcia-sierra@cell.cinvestav.mx; 4 Development and Neural Regeneration Laboratory, Cell and Molecular Biology Department, CUCBA, University of Guadalajara, Zapopan 45220, Mexico; gasca.mx@hotmail.com (Y.G.-M.); carlos.beas@academicos.udg.mx (C.B.-Z.)

**Keywords:** Alzheimer’s disease, erythropoietin, protective effects, neuroinflammation, apoptosis, oxidative stress, excitotoxicity

## Abstract

Substantial evidence in the literature demonstrates the pleiotropic effects of the administration of recombinant human erythropoietin (rhEPO) and its molecular variants in different tissues and organs, including the brain. Some of these reports suggest that the chemical properties of this molecule by itself or in combination with other agents (e.g., growth factors) could provide the necessary pharmacological characteristics to be considered a potential protective agent in neurological disorders such as Alzheimer’s disease (AD). AD is a degenerative disorder of the brain, characterized by an aberrant accumulation of amyloid β (Aβ) and hyperphosphorylated tau (tau-p) proteins in the extracellular and intracellular space, respectively, leading to inflammation, oxidative stress, excitotoxicity, and other neuronal alterations that compromise cell viability, causing neurodegeneration in the hippocampus and the cerebral cortex. Unfortunately, to date, it lacks an effective therapeutic strategy for its treatment. Therefore, in this review, we analyze the evidence regarding the effects of exogenous EPOs (rhEPO and its molecular variants) in several in vivo and in vitro Aβ and tau-p models of AD-type neurodegeneration, to be considered as an alternative protective treatment to this condition. Particularly, we focus on analyzing the differential effect of molecular variants of rhEPO when changes in doses, route of administration, duration of treatment or application times, are evaluated for the improved cellular alterations generated in this disease. This narrative review shows the evidence of the effectiveness of the exogenous EPOs as potential therapeutic molecules, focused on the mechanisms that establish cellular damage and clinical manifestation in the AD.

## 1. Introduction

**Figure 1:**
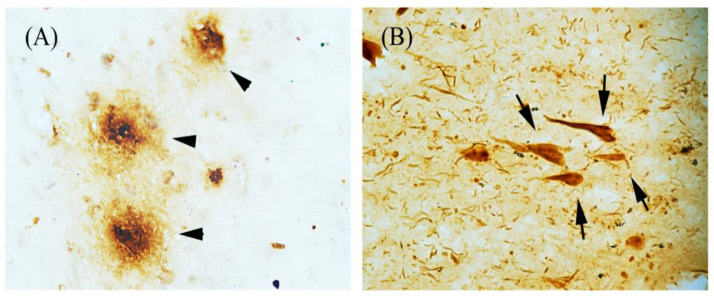
Characteristic hallmarks of Alzheimer’s disease (AD) brains. Bright-field immunohistochemistry microscopic images from hippocampus of an AD patient: (**A**) Amyloid beta plaques (arrowhead) and (**B**) Neurofibrillary tangles (arrows), formed by abnormal accumulations of Aβ and tau-p protein, as recognized by specific antibodies, respectively.

Alzheimer´s disease (AD) is a neurodegenerative disorder and the most common form of dementia in elderly people [1]. Clinically, AD is characterized by progressive mental decline and cognitive dysfunction, which have been associated with neuronal degeneration and excessive glial activation in brain areas that are mainly related to learning and memory, such as the hippocampus and neocortex [2,3,4]. The typical pathological hallmarks in patients with AD are abnormal deposition of amyloid-β (Aβ) plaques and accumulation of hyperphosphorylated tau (tau-p) protein in neurofibrillary tangles (NFTs) [5] (Figure 1). Aβ is generated from amyloid precursor protein (APP) by sequential proteolytic cleavage from both beta and gamma secretases [6]. The Aβ peptides form aggregates mainly in the extracellular space, but also within neurons and glial cells [7]. On the other hand, the tau hyperphosphorylation can occur due to an imbalance between kinase and phosphatase activities. The abnormal hyperphosphorylated state interferes with tau function, contributing to the disruption of intracellular transport and loss of synaptic contacts [8]. This tau-p state promotes its self-aggregation into fibrillary structures and increases cell toxicity due to its uncontrolled accumulation into the neuronal body [9,10,11]. In the AD, despite its uncertain origin, long process of evolution and the complexity of its study, it is known that Aβ and tau-p abnormal accumulation in the brain promotes different responses such as inflammatory, oxidative and excitotoxic that involve the activation of different signaling pathways that lead to neurodegeneration by aberrant apoptotic death. It is currently unknown whether abnormal protein aggregates in AD brains are the cause of the disease or a secondary phenomenon; unfortunately, this disease is a growing public health problem that lacks an effective therapeutic strategy for its treatment. To this day, there are only pharmacological drugs aiming to reduce the symptoms. However, research groups around the world are focusing their efforts on finding suitable methods for the prevention, early detection and possible cure for this disease. In the past few decades, researchers have redirected their attention towards erythropoietin (EPO), an endogenous molecule with a pleiotropic role, although not fully understood, in neurodegenerative diseases.

EPO is a small protein (30 kDa) [12] that is widely recognized as a hematopoietic factor, although it has been described as having other effects on the brain and other organs [13,14,15]. In the brain, the production of EPO and its receptor (EPOR) occurs in endothelial cells, astrocytes and neurons [15,16,17,18,19,20,21]. EPO is involved in neuroprotection, neurogenesis and regeneration, and acts as a messenger in autocrine or paracrine signaling [14,22]. Particularly, EPO gene expression can be regulated by hypoxia-inducible factor-1 (HIF-1) [20,23,24,25], which in turn is activated by multiple stressors [26,27]. In humans, under damage conditions in different neurological disorders, the EPO/EPOR system is upregulated in the brain tissue, and only in some cases in cerebrospinal fluid (CSF), as compared to healthy cases [15,28,29,30,31,32,33,34]. This system is considered an endogenous protective response to injury [30,31] that presumably acts in conjunction with vascular endothelial growth factor (VEGF) and other trophic factors [35,36,37]. Moreover, due to the ongoing cell degeneration, EPO/EPOR system upregulation in the brain has been suggested to be a normal response to aging, but not sufficient to prevent neurodegenerative disorders such as AD [13,18,28,32]. Interestingly, the works reviewed here provided evidence that the cellular protective effect of rhEPO and its molecular variants increase the cell viability in vitro models, which could occur through the decrease in load protein aggregates in vivo models as well as clinical trials, and their effects against pathological hallmarks are mediated by both Aβ and tau-p proteins, although the molecular mechanisms or signaling pathways involved are unclear yet. Regarding this, we think that the application of rhEPO or any of its molecular variants could strengthen the intrinsic EPO-mediated protection system, so the signaling mechanism involved, as well as doses, routes of administration, duration of treatments and application times, among others, which can influence and thus explain the variability in the reported results, are then important goals to investigate. 

## 2. Search Method and Criteria for Literature Inclusion

Medline via PubMed database (http://pubmed.ncbi.nlm.nih.gov) was searched to identify articles published up to May 2020 about rhEPO and its molecular variants applied as treatment to Alzheimer’s disease. The search keywords were “Erythropoietin treatment” or “EPO treatment” and “Alzheimer’s disease” or “AD”, as well as combination of these terms. Only research articles that reported the effects of rhEPO or any of its molecular variants in AD models (in vitro or in vivo), developed as a consequence of the exposition or expression of Aβ or tau-p, were included in this review. No limitations were set on in vivo studies regarding species or age. On the basis of the inclusion criteria, a total of 32 studies were included in this narrative review.

## 3. rhEPO and Molecular Variants

**Figure 2:**
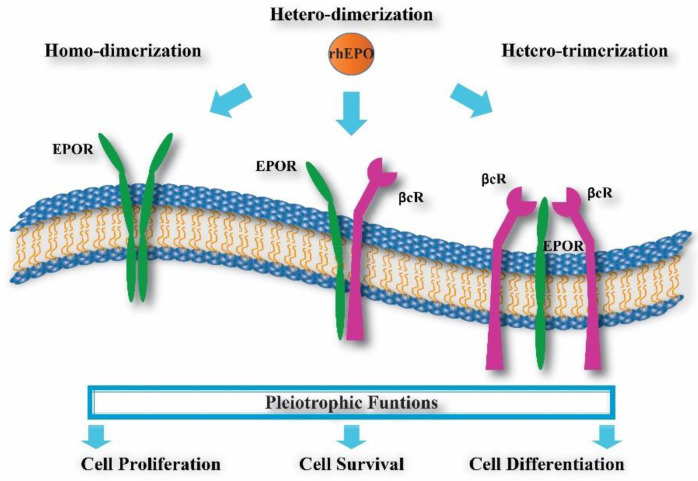
EPO receptors. The rhEPO has been proposed to interact with three possible receptor complexes in initiating its pleiotrophic effects: two units of EPOR = homodimer (green structure), one EPOR and one βcR units = heterodimer (green and purple structure) or one EPOR plus two βcR units = heterotrimer (green and purple structures).

Different research groups have increased their interest in studying the effect of rhEPO administration as a treatment for neurological diseases such as AD [38,39,40]. Evidence from different reports indicates that rhEPO can cross the intact blood–brain barrier (BBB) [16,22] and preserve its biological activity [41,42]. Through rhEPO interaction with homodimeric, heterodimeric or heterotrimeric EPORs (Figure 2) [43,44,45], rhEPO administration can induce neurotrophic effects, thereby promoting differentiation, cellular protection and regeneration [14] against brain injuries and degeneration [13,19,22,46,47,48], although the mechanism used is still unclear. 

**Figure 3:**
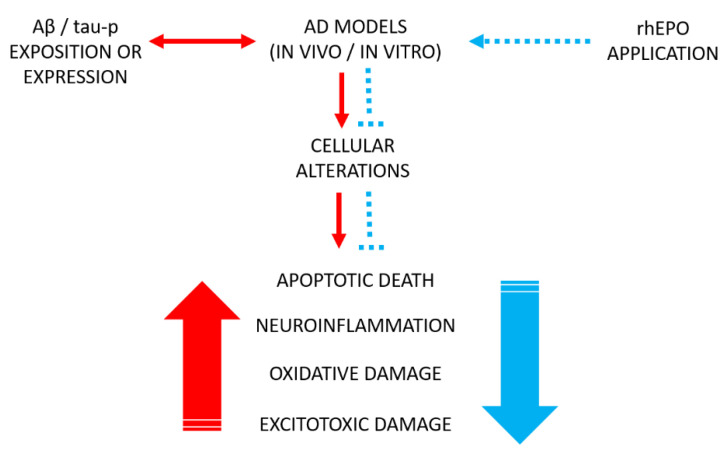
rhEPO effects on cell damage in AD models. The rhEPO application as a pharmacological treatment (blue dotted arrow) diminishes cellular alterations (blue dotted lines) generated in response to exposition or expression to Aβ or tau-p proteins in vitro and in vivo AD models (double-head red arrow). Red big arrow refer to increased cellular alterations, while blue big arrow refer to damage inhibition.

Even so, both in vitro (cell culture) and in vivo (animal models) experimental studies strongly support the protective role of rhEPO [47,49,50] (Figure 3), although, to date, there is little evidence for AD patients or other neurological diseases. However, something that has to be considered is that chronic application of rhEPO has multiple adverse side effects. To reduce or prevent these effects, chemical changes in the rhEPO molecule have been assessed, and new variants with different experimental results have emerged.

Molecular variants of rhEPO include asialoerythropoietin (Asialo-EPO), a deglycosylated form of rhEPO, which, in experimental studies, has shown an effect on neuroprotection and few changes in hematocrit due to its short half-life [49,51,52]. Carbamylated EPO (CEPO) is a conformationally modified variant of rhEPO that replaces lysines with homocitrullines, which is nonerythropoietic, but maintains the tissue-protective effect; interestingly, it is not recognized through EPOR classical interaction [44,53,54]. EPOL is a variant of the rhEPO molecule, which has a different glycosylation pattern (low sialylated bi-antennary structures) and no hematopoietic activity, but has a protective activity against oxidative stress [55,56]. Neuro-EPO is another variant with low sialic acid content and a short half-life. This variant has no hematopoietic effects, but exhibits neuroprotective effects when administered intranasally (i.n.) [40,57,58]. On the other hand, an rhEPO analogue called cTfRMAb-EPO, obtained by fusing EPO with a rat/mouse chimeric monoclonal antibody targeting the transferrin receptor (TfR1), acts as an efficient neuroprotective agent with minimum effects on the hematocrit (compared with rhEPO) when administered intraperitoneally (i.p.). TfR1 is expressed in the brain capillary endothelium and functions as a receptor-mediated transcytosis system. Thus, its ligands can be used for non-invasive delivery of drugs to the brain via the transvascular route. TfR1 can be recognized by the monoclonal antibody against TfR1, which binds to a different site than that of transferrin, and is an alternative drug delivery vector. This action crosses the BBB and leads to widespread drug delivery throughout the brain owing to the dense network of the cerebral vasculature. Interestingly, the cTfRMAb-EPO fusion protein also binds to EPOR with high affinity [59]. All of these rhEPO variants have been demonstrated to have different degrees of effectiveness when are administered in the AD experimental models (Table 1). The effects seem to depend on several things, such as the doses, route of administration, application times and duration of treatments, but are also related to the kind of receptor and its recognition. 

## 4. Effects of rhEPO and Some Its Molecular Variants in Experimental Models of Alzheimer’s Disease

In AD, an uncontrolled increase in neurodegeneration that affects the brain regions involved in memory and learning has been reported, with the hippocampal cells the most vulnerable to damage [9,42,60,61,62]. The cell damage has been mainly associated with the abnormal presence or accumulation of Aβ species directly related to apoptosis by promoting the exposure of phosphatidyl-serine to the outer membrane, caspase activation and glial overactivation [15,63,64], as well as with the formation of a pore in the cell membrane, which allows the massive entry of cations (e.g., Ca^2+^), altering ion homeostasis and energy balance. These changes generate the mis-sorting of tau-protein in the cell body, cell dysfunction and apoptosis, as demonstrated by experimental evidence [65,66,67,68,69], characteristics that have been related to the early alterations in cognitive function [70].

**Figure 4:**
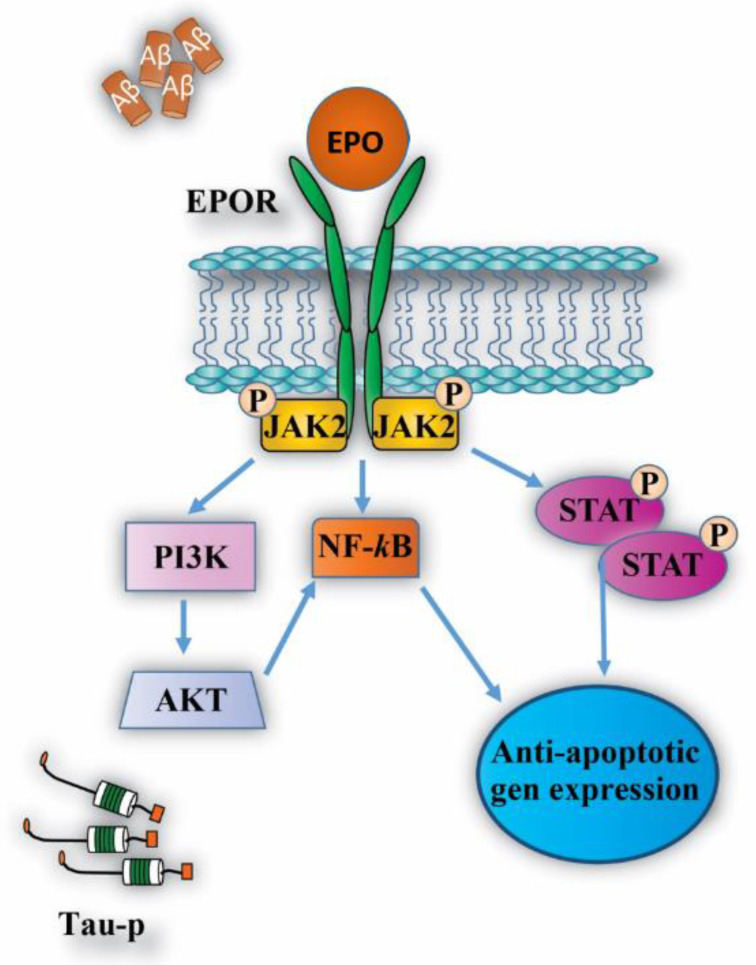
Intracellular signaling pathways activated by EPO/EPOR system. EPO molecule activates different intracellular pathways through EPOR, leading to anti-apoptotic gene expression as well as the inhibition of pro-apoptosis genes. These actions allow for cell proliferation, differentiation and cell survival against the pathological stimulus generated by Aβ and tau-p protein in AD.

Therefore, through to use of diverse AD-models both in vitro and in vivo, the effects of molecules with pleiotropic activity such as rhEPO and its molecular variants have been evaluated as a therapeutic agent for this disease. One of the most studied effects of the EPO molecule is neuroprotection through anti-apoptotic mechanisms [40,45,63,71,72,73,74,75,76,77], regulated by different signaling pathways such as JAK/STAT [78], among others (Figure 4) [49,55,56,79,80,81]. In addition, the inhibition of inflammation, oxidative stress and excitotoxicity appear to be associated with the activity of EPO/EPOR in the central nervous system (CNS). 

### 4.1. Neuroprotective Effects

According to the evidence in vitro, using PC12 and SH-SY5Y cell lines as well as primary cultures of hippocampal neurons, the exposure to Aβ peptides or fragments like Aβ25-35 (short toxic variant), induces degenerative changes such as oxidative stress and high levels of tau-p and apoptosis [82,83], while a dose-dependent decrease in both apoptotic cell death and other alterations was reported when cell cultures were pre-treated or co-incubated with rhEPO [56,63,79,84,85,86].

Li et al. [63] reported in PC12 cell culture that pre-treatment with different concentrations of rhEPO (0.5–10 IU/mL) one hour before exposure to Aβ25-35 peptide reduces the number of apoptotic cells through an increase in Bcl-2 expression and decrease in both Bax expression and caspase-3 activity. This result supports other studies where the anti-apoptotic effects of rhEPO are presumably due to the increased expression of apoptosis inhibiting genes such as Bcl-2, Bcl-xL, XIAP and c-IAP2 [85,87]. Furthermore, in a comparative study, the protective effects of rhEPO and EPOL were tested in PC12 cell cultures. Cell cultures subjected to chronic exposure to Aβ40 were incubated with rhEPO or EPOL at different concentrations (10, 50 and 100 ng/mL). Primary cultures exposed to Aβ40 as well as organotypic cultures exposed to Aβ25-35 (both from rat hippocampus) incubated with rhEPO or EPOL in a unique concentration (100 ng/mL), respectively, were also alternately used. The results showed lower levels of cell death, detected by MTT or propidium iodide fluorescence assays, in EPOs-treated cultures; although EPOL treatment (50 or 100 ng/mL doses) was effective in all culture experiments, rhEPO (100 ng/mL dose) failed to provide protection in organotypic cultures [56,86].

Experimental evidence in SH-SY5Y cell cultures demonstrates that exposure to Aβ25-35 increases the level of tau-p protein, which is attributed to the over-activation of glycogen synthase kinase-3β (GSK-3β), which diminished when the cultures were pretreated for one hour with different doses of rhEPO (5, 10 or 20 IU/g), and thereby significantly reduced the percentage of cell death [84,88]. Different authors suggest that the cellular protective effect of rhEPO, mediated by suppressing tau phosphorylation, occurs via PI3K/AKT-GSK-3β signaling [48,84,88,89,90,91]. In addition, reports in primary neuronal cultures from rats suggest that both the induction and redistribution of tau-p as well as its accumulation in the somatodendritic compartment, caused by Aβ oligomers and inhibition of protein degradation, increases cytotoxicity [67,92]. Interestingly, in some in vitro assays, the protective effect of rhEPO under Aβ damage has also been associated with its amino acid composition, as the EPO molecule has a hydrophobic core, which can interact with Aβ in the C-terminal region, important for Aβ/Aβ peptide interaction, and insertion into the cell lipid membrane [56]. This action is similar to that proposed for neuroprotective agents, which prevented Aβ aggregation [93]. Currently, there is more experimental evidence in vitro on the inhibition of the Aβ effect by rhEPO (Table 2), so we emphasized that more attention should be given to the participation of the tau-p aggregates and evaluating the protective role of rhEPO against the cellular effects generated by them. 

Concomitantly, in vivo models of AD have been used to characterize the neuroprotective effects of rhEPO and its molecular variants, as well as to determine the optimum dose, route of administration, treatment duration and application times, and adverse side effects. Intracerebroventricular (ICV) injection of a diabetogenic substance called streptozotocin (STZ) in rodents is used to generate a valid model of sporadic AD. STZ in experimental subjects causes long-term and progressive deficits in learning, memory, and behavior [95,96,97] as a consequence of neuronal loss associated with metabolic alterations, since cerebral energy deficit mainly occurs in the hippocampus and dentate gyrus (DG) [41,98,99]. The i.p. administration of rhEPO (5000 IU/kg), every other day for 2 weeks in the ICV-STZ model, induced neurogenesis in DG and protected brain cells from STZ-induced damage, thereby improving memory deficits without presenting significant erythropoietic alterations [41,97].

Importantly, similar effects were obtained in a comparative study between the administration of rhEPO and Neuro-EPO in an AD model generated by ICV injection of Aβ25-35 in non-transgenic mice (ICV-Aβ25-35 model) [40,100]. Neuropathological hallmarks, such as reactive gliosis, caspase over-activation, oxidative stress, neuronal loss and memory deficits, were reported in this AD mouse model. The administration of each of the evaluated EPO molecules at different doses, prevented neuronal loss in the hippocampal CA1 region, and reduced the number of brain amyloid plaques, improving learning deficits [40,101]. In the same way, the protective effect of both molecules was evaluated in the transgenic mice model Tg2576 developed by Hsiao et al. [102], which overexpresses human APP (Swedish double mutation). This mouse at 12 months of age showed the first signs of Aβ deposits in plaques and, in parallel, developed behavioral deficits, particularly in spatial memory. In this study, the i.p. administration of rhEPO (5000 IU/kg) for five days improved contextual memory and synaptophysin expression, and reduced endothelial dysfunction and the amount of Aβ plaque in the brain [72], while Neuro-EPO treatment (125 or 250 IU/kg, three times a week) for 2 months reduced apoptotic brain damage caused by Aβ accumulation, as well as neuroinflammation, synapse loss and declines in long-term potentiation [101]. In both studies, even at low doses, Neuro-EPO treatment had better protective effects than rhEPO [40,57,101], without hematopoietic effects [57,58]. Other reports have suggested that Neuro-EPO treatment may block both intrinsic and extrinsic apoptotic pathways by modulating the Bax/Bcl-2 ratio, reducing caspase-3 activity in response to Akt activation in a PI3K-dependent manner, and decreasing the abnormally increased levels of TNF-α or FasL [40,101]. Furthermore, Rodriguez-Cruz et al. [101] reported a decrease, in the load of Aβ species, of the 6E10 antibody (specific to Aβ1-16) in the hippocampus and cortex after Neuro-EPO treatment.

Another comparative study between rhEPO and CEPO was realized in a double transgenic mouse model of AD (AβPP/PS1) developed by Borchelt and his colleagues, that expresses the APP Swedish mutation and the mutant human presinilin 1 (PS1-dE9) [103]. This model presents an increase in Aβ plaque in the brain, microglial over-activation, synapse loss and decreased spatial memory. The study showed that both rhEPO and CEPO treatments improved mice memory and health conditions without affecting erythropoiesis. However, only rhEPO decreased the amount of Aβ plaques and soluble Aβ species in the brain, unlike CEPO, suggesting that EPO molecules may have different effects on molecular substrates [104]. Finally, in the same AβPP/PS1 transgenic mouse model, cTfRMAb-EPO (3 mg/kg b.w, i.p.) was administered three days a week for eight weeks; this induced the reduction in the load of Aβ plaque as well as microglial activation, improving synaptic activity and spatial memory [59].

Interestingly, data obtained from animal models for the AD show that exogenous EPO treatment reduces cognitive problems and improves behavior as a consequence of reduced neuronal damage and cell death caused by abnormal protein aggregates of Aβ and tau-p, which also diminish [63,84,105]. In summary, the effectiveness of rhEPO and its molecular variants’ administration in animal AD models depends on the route and time of administration, as well as the dose at which it is administered, points that have to be considered in the interpretation of results. 

### 4.2. Anti-Inflammatory Effects

Neuroinflammation is an important component in the pathogenesis and progression of AD and other neurological diseases [41]. Aβ plaques and NFTs, the major pathological hallmarks of AD, have been associated with neuroinflammatory reactions leading to negative physiological responses, such as massive glial activation, synaptic dysfunction, mitochondrial damage and apoptosis, which are all signs of degeneration [2,3,40,101,106]. According to reports in experimental models of brain diseases, rhEPO promotes a decrease in both glial response and pro-inflammatory cytokine levels in brain tissue, which are part of its anti-inflammatory effects [21,53,76,107,108,109]. 

In patients and animal models of AD, over-activation of glial cells in the brain is a sign of neuroinflammation and cell damage [106,110,111]. Activated glial cells release large amounts of pro-inflammatory and neurotoxic cytokines, such as TNF-α, IL-1β, IL-6, IL-8 and monocyte chemoattractant-1 (MCP-1) [109,112,113] in addition to free radicals (e.g., reactive oxygen species (ROS) or nitric oxide (NO)), which directly increase apoptotic cell death [114]. TNF-α is a key cytokine that initiates the inflammatory cascade. In the healthy adult brain, it is synthetized at low levels by microglia and neurons [110,115]. However, high levels of TNF-α have been reported in the AD brain. Studies suggest that this alteration promotes Aβ production through the over-activation of β-secretase and, in turn, increases tau-p levels due to GSK-3 β activity. The accumulation of these altered proteins triggers chronic inflammation, neuronal death and, in consequence, cognitive dysfunction during the progression of AD [110,116,117,118,119,120].

Animal models have been used to study the relationship between TNF-α and both Aβ and tau-p in AD [110,121,122]. For example, in a non-transgenic mouse model of AD generated by hippocampal injection of Aβ40, the inhibition of TNF-α by treatment with adalimumab (1 mg/kg, i.p.), an anti-TNF-α drug, reduced the injuries triggered by the Aβ peptide, contributing to the reestablishment of cognitive function in experimental subjects [110]. Another study suggested that adalimumab inhibits the inflammatory response because it binds to TNF-α and blocks its receptor binding [123]. Contrary to the inhibition of TNF-α as a protective effect, it has been also suggested that the microglial phagocytic activity of Aβ peptides may be mediated by TNF-α, and its inhibition could be responsible for the increase in Aβ aggregates, as well as the increase in cognitive alterations [124,125]. The dual role of TNF-α has been correlated with differences in recognition by its receptor [126]. On the other hand, the increased levels of TNF-α, IL-1β, IL-6, IL-8 and other cytokines, as well as decreased brain choline acetyltransferase (ChAT) activity in the ICV-STZ rat model, were reverted after a dose of rhEPO (5000 IU/kg a day; i.p.), demonstrating its anti-inflammatory role [41,97,99,117]. Maurice et al. [40] reported similar data in a comparative study between rhEPO (250 µg/kg; i.p.) and Neuro-EPO (125 µg/kg; i.n.) in the ICV-Aβ25-35 AD mouse model. Both treatments significantly prevented the increased levels of TNF-α and IL-1β induced by the injection of Aβ25-35 peptide. The anti-inflammatory effects of Neuro-EPO were also corroborated in a study with aged transgenic mice (Tg2576). The brain of these mice presents an intense inflammation and glial over-activation due to increased Aβ deposits and plaques, which were decreased significantly after Neuro-EPO treatment (125 and 250 µg/kg; i.n.) [101]. These results are in agreement with previous studies that used rhEPO and/or Neuro-EPO as an inhibitor of the immune response to damage, in different experimental animal models [41,53,97,99,101,117,127].

In other reports, it has been suggested that rhEPO may also prevent microglial over-activation generated in response to Aβ toxicity, through of the regulation of EPOR signaling by the Wnt1/PI3-K1/mTOR pathway, which leads to the inhibition of NF-kB p65, which prevents its nuclear translocation and allows the expression of an anti-apoptotic gene such as Bcl-xL, as well as suppressors of TNF-α, IL-6 and IL-1β [85,110,112,113,128], thereby directly altering the reactive state of the cell and exhibiting anti-inflammatory effects [129,130,131]. Interestingly, studies have been reported that EPOR expression is regulated by pro-inflammatory cytokines [115,132]. Studies suggest that upregulation of EPOR in glial cells under AD may facilitate the action of exogenous EPO molecules, thereby protecting neuronal cells from damage generated by Aβ, pro-inflammatory cytokines and ROS [15,133]. 

### 4.3. Anti-Oxidant and Anti-Excitotoxic Effects

Both oxidative and excitotoxic damage are common events in AD, which leads to mitochondrial injury, genomic DNA alterations and other processes that generate cell death in susceptible brain regions such as the hippocampus [134,135,136,137,138]. The protective effects of rhEPO against different stressor molecules (e.g., Aβ species and Glu) that promote oxidative stress and excitotoxicity have been proven in several experimental models [20,63,84,139,140]. For example, exposure of PC12 cell cultures to Aβ25-35 peptides generated oxidative stress as result of increased ROS production, altering mitochondrial membrane phospholipids and causing apoptotic cell death. However, pre-incubation (one hour) at different concentrations of rhEPO (0.5–10 IU/mL) reduced these abnormal effects generated by Aβ25-35 peptides, but had the maximum protective effects achieved with 2 IU/mL [63]. In the same cell model, in a comparative study between rhEPO and EPOL, Castillo et al. [56] reported that oxidative stress induced by chronic exposure to Aβ40 was avoided with pre-treatment of rhEPO (100 ng/kg) or EPOL (50 ng/kg). This effect is associated with an increase in the expression of the anti-apoptotic gene Bcl-2 [55,56]. In the same study, but in rat hippocampal organotypic cultures treated with Aβ25-35, the increase in ROS levels observed in the CA1 brain region, were reestablished to nearly normal levels when the cells were co-incubated with rhEPO or EPOL. In this case, better results were obtained with EPOL even at low concentrations [56]. These results are consistent with other studies using primary hippocampal and cerebral cortex cultures from rats. In these studies, Glu-induced neuronal death or hypoxia decreased dramatically with rhEPO pre-treatment in a dose-dependent manner [19].

On the other hand, in the ICV-STZ rat model, the administration of rhEPO (5000 IU/kg; i.p.) decreased the oxidative damage (increased inflammatory cytokines and mitochondrial dysfunction) generated by STZ injection [41]. Similar observations were reported in an ICV-STZ mouse model treated with rhEPO (500 and 1000 IU/kg; i.p.), improving memory deficits and oxidative stress in the brain [141]. Maurice et al. [40], in hippocampal extracts from ICV-Aβ25-35 mice, reported an increase in the levels of membrane lipid peroxidation. The authors showed that the administration of rhEPO (250 µg/mL; i.p.) and Neuro-EPO (125 µg/mL; i.n.) significantly reduced the oxidative effects, even though Neuro-EPO used lower doses [40]. Finally, these results were also corroborated using aged Tg2576 mice treated with Neuro-EPO (125 µg/mL; i.n.). According to the study, abnormally increased levels of membrane lipid peroxidation in hippocampal cells, in response to Aβ-induced oxidative stress, were significantly reduced [101]. In both experimental cases, the survival rate of the mice increased, as well as their learning ability [40,101]. 

Although the antioxidant mechanism of EPO in AD brains is still unknown, research groups have suggested that it promotes NF-kB p65 nuclear translocation and prevents Akt phosphorylation generated in response to damage induced by Aβ accumulation. These actions could help stabilize the mitochondrial membrane potential (Bcl-2/Bax ratio) and attenuate ROS production [57,63,85,94,142]. In addition, experimental evidence points to the possibility of an increased activation of antioxidant enzymes such as catalase, superoxide dismutase, or glutathione peroxidase, with rhEPO treatments [38,143,144,145,146], or that EPO could act directly as an ROS scavenger [147,148]. Interestingly, the antioxidant effect of exogenous EPO molecules apparently does not alter the intracellular redox condition [94].

Along with oxidative stress, excitotoxicity has been considered as a cause or consequence of neuronal damage in many neurodegenerative disorders [149,150,151,152]. Usually, excitotoxicity is described as the abnormal extracellular Glu accumulation, which over-activates its own receptors’ (GluR) promoted event activation, such as oxidative stress and mitochondrial dysfunction, that are conducive to cell death [153,154,155]. It is well known that cell injury causes an increase in intracellular Ca^2+^ levels, which, in turn, promotes a greater release of Glu from the vesicles. Like a vicious circle, Glu excitotoxicity may induce an increase in intracellular free radicals and Ca^2+^, thereby activating both pro- and anti-apoptotic responses in cells; depending on the duration and severity of the injury, the cell either survives or dies [156]. In a study in which cultured cortical neurons and astroglial cells were exposed to hypoxia accompanied by glucose deprivation damage, rhEPO treatment (30 pM) showed protective effects on neuronal cells, but not on astroglial cells [157]; these data are consistent with previous findings [158]. Although the astrocyte collects extracellular Glu released by neurotransmission through specific transports to prevent excitotoxicity, Glu-transports have been reported to decrease in the AD brain. This decrease has been associated with abnormal APP expression [159,160].

It has been postulated that the increase in both Glu and Aβ in the AD reduces the anti-apoptotic Bcl-2 and increases the expression of pro-apoptotic Bax protein. This action releases cytochrome C protein into the cytosol and increases intracellular Ca^2+^ levels. Consequently, intrinsic apoptotic signaling is activated [161,162]. According to the published data in PC12 cell cultures exposed to Aβ species and rhEPO (2 IU/mL) treatment, mitochondrial membrane integrity was maintained to increase Bcl-2 and reduce Bax expressions, thereby maintaining the Bcl-2/Bax ratio [63]. Garzon et al. [94] in primary neuronal cultures exposed to Glu excitotoxicity and Neuro-EPO (100 ng/mL) reported that morphological changes (cell body shrinkage, formation of blebs on the cell surface, pyknotic nucleus and loss of dendritic process) and apoptotic cell death were reduced with Neuro-EPO treatment, even 15 min after exposure to Glu [94]. Castillo et al. [56], using primary hippocampal neuronal cultures exposed to Aβ40 and co-incubated with EPOL (100 ng/mL), reported that the treatment could preserve the level of presynaptic proteins, such as synaptic vesicle protein 2 (SV2), which are related to neurotransmitter release and can be altered by abnormal Ca^2+^ levels resulting from Aβ toxicity. All of these data suggest that rhEPO or its molecular variants play a protective role by regulating the release of neurotransmitters; however, further studies are needed to elucidate the mechanism by which this regulation occurs. In summary, from the analysis of these studies (in vivo) to date (Table 3), Neuro-EPO administered via i.n. has proven to be the most effective in the treatment or prevention of AD pathophysiology.

## 5. rhEPO Evidence in Human Trials with Alzheimer’s Disease

As we explained before, the neuroprotective effect of rhEPO has been confirmed in different experimental models, however, the evidence in patients with cognitive impairment is scarce [46,74,163,164,165,166,167]. Some existing reports indicate that the level of EPO in the brain and CSF may increase with aging [28,32]. However, EPO levels in AD patients have been reported to be low, while EPOR levels are high [15]. This is considered a failure of the intrinsic EPO/EPOR protection system due to a shortage of cytokines [75,142]. EPO is an important molecule for cell protection in different pathologies; therefore, its use is being evaluated in clinical trials [13,39,43,146,168,169,170,171]. 

Unfortunately, there are few clinical studies on the effects of rhEPO administration in patients with AD, which have focused on the treatment of dementia symptoms and scarcely diminish the presence of characteristic hallmarks (Aβ and tau-p aggregation) of this pathology [172,173,174,175]. Even so, preliminary studies have shown that rhEPO administration in AD patients can help regulate the elevated levels of Aβ and tau-p proteins. They also demonstrated tissue protection and behavioral improvement in most cases [74,142,175,176]. Although there is no clear evidence of the anti-apoptotic effect of rhEPO in clinical trials with AD, other pathological studies involving Aβ or tau proteins also show positive results. For example, data obtained from a study of human neonates with hypoxic-ischemic encephalopathy (HIE) showed that elevated serum levels of tau were clearly reduced to nearly normal conditions after rhEPO treatment (200 IU/kg, intravenously) once a day for ten consecutive days [146]. Similarly, clinical studies in patients with chronic kidney disease (CKD) and cognitive dysfunction reported that abnormally elevated levels of Aβ, GSK3-β and tau-p, among others, were reestablished after treatment with rhEPO (100 IU/kg) administered twice weekly for 6 months [134]. These findings suggest that rhEPO improves the neurological outcome of neonatal behavior and enhances the neuropsychological assessment of adult patients, respectively [134,146]. Finally, although there is little evidence on the anti-inflammatory effects of rhEPO in AD patients, some clinical studies have reported that the inhibition of pro-inflammatory molecules such as TNF-α [177], the increase in HIF [142], and even some related effects with exogenous EPO molecules, could be considered as potential treatments for neurodegenerative diseases [22,76,178]. However, some of the data obtained are refuted due to differences in human clinical trials, such as differences between experimental protocols (application route, concentration and number of rhEPO doses), patient inclusion criteria (differential diagnosis of disease and age), small study groups, short follow-up periods, and interpretation of results. This situation, among others, increases the variability of the results, therefore, they must be carefully analyzed.

## 6. Conclusions

**Figure 5:**
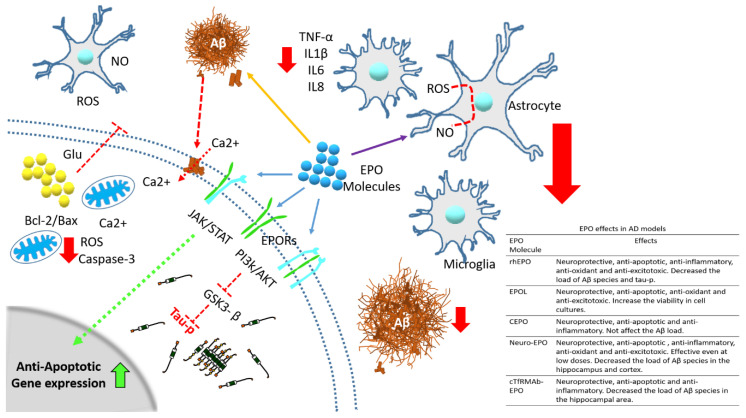
Protective effects of rhEPO and its molecular variants against the damages produced by the abnormal accumulation of proteins (Aβ and tau-p) analyzed in different models of AD. The rhEPO or its molecular variants through of the interaction with its receptor (different complex of them)(blue arrows) they activate molecular pathways (such as PI3K/AKT or JAK/STAT) responsible for decreasing the abnormal cellular processes such as apoptotic gene expression (dotted green arrow and solid green arrow into nucleus), caspase-3 activation (red arrow), release of Glu (dotted red line), over activation of GSK3-β (dotted red line) all them inside the cell and cytokines production (red arrow) outside of cell, among others, associated with cellular damages by apoptosis, oxidative stress and excitotoxicity. In addition, have been proposed that the exogenous EPO molecules also may avoid the aggregation of Aβ (yellow arrow) and tau-p through to join with them and decreasing their load in brains during AD progression. The pathological mechanism in this disease also involve the participation of glial cells, responsible to increase the level of free radical and pro-inflammatory cytokines and inducing neuronal cell death, although they are also regulated by the EPO/EPOR protective system (purple arrow). Red big arrows refer to decreased cellular alterations by EPO treatment.

Researchers in the field of neuroscience are increasingly proposing that the protective effects of rhEPO and its molecular variants could be used as an alternative to treat neurodegenerative disorders as AD itself. According to the data described in this narrative review, the reinforcement of the endogenous EPO/EPOR system by exogenous EPO molecules may be crucial in protecting the brain against neurological damage caused by abnormal Aβ and tau-p aggregates or other pathological stimuli that promote the neurodegeneration in AD (Figure 5). This is based on extensive evidence that the EPO molecule can act directly or indirectly over abnormal protein aggregates, avoiding their formation and diminishing their load during disease progression. However, the mechanisms that promote and regulate the protective effect (anti-apoptotic, anti-inflammatory, antioxidant and anti-excitotoxic) in the AD models are not totally clear. For this and other reasons, the EPO/EPOR system deserves further investigation to better understand its role as pharmacological agent. Finally, we believe that pleiotropic function of rhEPO and its molecular variants in the CNS, not only for neurological diseases, can be explained through functional receptor-mediated differential cellular response and not only by the kind of EPO molecule. For these reasons, studies should be directed towards the contributions of EPORs, since the expression of specific types of receptors determines the cellular response, although it may not only be receptor- or cell-dependent, but also subject to the pathophysiological conditions found in the brain of patients undergoing any neurological disease such as AD.

## Figures and Tables

**Table 1:** Molecular variants of rhEPO. Asialoerythropoietin (Asialo-EPO); Carbamylated EPO (CEPO); EPOL; Neuro-EPO and an EPO analogue called cTfRMAb-EPO. All them have low or no hematocrit effects but have protective effects preserved. As reference of chronic treatment, rhEPO was included.

Epo Molecule	Molecular Modification	Hematocrit Effects	Protective Effects	References
rhEPO	None	High	Yes	[22,13]
Asialo-EPO	Deglycosylated form of EPO	Low	Yes	[51,52]
CEPO	Conformational modification (replaces lysines with homocitrulines)	Low	Yes	[54]
EPOL	Different glycosylation pattern (low sialylated bi-antennary structures)	None	Yes	[55,56]
Neuro-EPO	Low sialic acid content	None	Yes	[40,57]
cTfRMAb-EPO	Fusion of chimeric antibody with EPO	Low	Yes	[59]

**Table 2:** In vitro AD models.

In Vitro Model	Noxious Stimulus	EPO Molecule	Timing and Doses of Administration	Effects	References
PC12	Aβ40 Oligomers Aβ25-35 peptide	rhEPO or EPOL rhEPO	Pre-treatment for 1 h or co-incubation for 24 h; doses used 10, 50 or 100 ng/mL Pre-treatment for 1 h; doses used 0.5-10 IU/mL	- Increased antiapoptotic gene (Bcl-2) expression - Avoided oxidative stress - Improved cell viability - Reduced the number of apoptotic cells - Increased Bcl-2 expression and decreased both Bax expression and caspase-3 activity - Maintained the integrity of the mitochondrial membrane - Increased cell viability	[56,63,79]
SH-SY5Y	Aβ25-35 Tau-p	rhEPO	Pre-treatment for 1 h; used doses 5, 10 or 20 IU/g	- Reduced the percentage of cell death - Decreased tau-p levels by inhibition of GSK3-β - Reversed the apoptotic effect and maintained cell viability	[84,88]
Primary culture of hippocampal neurons	Aβ40 oligomers Tau-p	rhEPO or EPOL	Co-incubation for 24 h; dose used 100 ng/mL	- Reduced oxidative stress and spontaneous intracellular Ca2+ oscillations - Increased antiapoptotic gene (Bcl-2) expression - Increased cell viability - Preserved the level of presynaptic proteins, such as SV2 - Reduced the Glu excitotoxicity	[55,56,94]
Organotypic rat hippocampus culture	Aβ25-35	rhEPO or EPOL	Co-incubation for 4 days; dose used 100 ng/mL	- Reduced ROS levels - Reduced glial activation - Increased antiapoptotic gene (Bcl-2) expression - Increased cell viability with EPOL but no with rhEPO	[56]

**Table 3:** In vivo AD models. ICV-STZ; ICV-Aβ25-35 = Intracerebroventricular injection models; Tg2576*; AβPP/PS1* = Transgenic models. rhEPO; EPOL; CEPO; Neuro-EPO and cTfRMAb-EPO = Molecular variants of EPO. i.p.—intraperitoneal; i.n.—intranasal = Administration route.

In Vivo Model	Noxious Stimulus	EPO Molecule	Doses and Route of Administration	Effects	References
ICV-STZ (rat)	Injection of STZ	rhEPO	Dose used 5000 IU/kg, every other day for 2 weeks; i.p	- Prevented apoptosis - Induced neurogenesis - Improved memory deficits - Reverted the increase of proinflammatory cytokines - Reversed the oxidative damage (mitochondrial dysfunction)	[41,97]
ICV-Aβ25-35 (mouse)	Injection of Aβ25-35	rhEPO or Neuro-EPO	Dose used 1250, 2500 and 5000 UI/kg once a day or 62, 125 and 250 IU/kg three times a day; i.p and i.n (respectively)	- Neuronal loss in the hippocampal CA1 region is prevented - Improved learning deficits - Reduced the number of brain amyloid plaques - Neuro- EPO treatment blocks both intrinsic and extrinsic apoptotic pathways - Prevented the increased levels of TNF-α and IL-1β - Reduced the oxidative effects (membrane lipid peroxidation)	[40,101]
Tg2576* (mouse)	Overexpression of human APP (Swedish double mutation)	rhEPO or Neuro-EPO	Dose used 5000 IU/kg once a day for five days or 125 and 250 IU/kg, three times a week for 2 months; i.p and i.n (respectively)	- Improved contextual memory and synaptophysin expression - Reduced endothelial dysfunction - Reduced the amount of Aβ plaques in the brain - Reduced glial activation - - Reduced the oxidative effects (membrane lipid peroxidation)	[72,101]
AβPP/PS1* (mouse)	Expression of human APP and mutant human presinilin 1 (PS1-dE9)	rhEPO or CEPO cTfRMAb-EPO	Dose used 2500 IU/kg or 2500 and 5000 IU/kg administered three days a week for four weeks respectively; i.p Dose used 3 mg/kg administered three days a week for eight weeks; i.p	- Improved mice memory and health conditions - rhEPO decreased the amount of Aβ plaques and soluble Aβ species in the brain, but not CEPO - Reduced the load of Aβ plaques and microglial activation - Improved synaptic activity and spatial memory	[104,59]
